# Cascade Transformations of 1-R-Ethynyl-9,10-anthraquinones with Amidines: Expanding Access to Isoaporphinoid Alkaloids

**DOI:** 10.3390/molecules26226883

**Published:** 2021-11-15

**Authors:** Sergey Francevich Vasilevsky, Ol’ga Leonidovna Krivenko, Irina Vasilievna Sorokina, Dmitry Sergeevich Baev, Tatyana Genrikhovna Tolstikova, Igor V. Alabugin

**Affiliations:** 1 V.V. Voevodsky Institute of Chemical Kinetics and Combustion, Siberian Branch of the Russian Academy of Science, 630090 Novosibirsk, Russia; marisha@kinetics.nsc.ru; 2 N.N. Vorozhtsov Novosibirsk Institute of Organic Chemistry, Siberian Branch of the Russian Academy of Sciences, 9 prosp. Acad. Lavrent’eva, 630090 Novosibirsk, Russia; sorokina.irina55@gmail.com (I.V.S.); mitja2001@gmail.com (D.S.B.); tg_tolstikova@mail.ru (T.G.T.); 3 Department of Chemistry and Biochemistry, Florida State University, Tallahassee, FL 32306, USA

**Keywords:** alkynes, amidines, cyclization, 2-R-7*H*-dibenzo[*de,h*]quinolin-7-ones, anti-inflammatory activity

## Abstract

The interaction of acetamidine and phenylamidine with peri-R-ethynyl-9,10-anthraquinones in refluxing n-butanol leads to the formation of cascade transformations products: addition/elimination/cyclization―2-R-7*H*-dibenzo[*de*,*h*]quinolin-7-ones and(or) 2-R-3-aroyl-7*H*-dibenzo[*de*,*h*]quinolin-7-ones. The anti-inflammatory and antitumor properties of the new 2-R-7H-dibenzo[*de,h*]quinolin-7-ones were investigated in vivo, in vitro, and in silico. The synthesized compounds exhibit high anti-inflammatory activity at dose 20 mg/kg (intraperitoneal injection) in the models of exudative (histamine-induced) and immunogenic (concanavalin A-induced) inflammation. Molecular docking data demonstrate that quinolinones can potentially intercalate into DNA similarly to the antitumor drug doxorubicin.

## 1. Introduction

Finding general correlations between molecular structure and reactivity is one of the central goals of organic chemistry. Previously, we found that reactions of 1-(R-ethynyl)-9,10-anthraquinones and multifunctional reagents (hydrazine, [1] guanidine, [2] urea, [3] thiourea, [4] ethylenediamine [5]) are very sensitive both to the reaction conditions and to the nature of substituents at the acetylene moiety.

A characteristic structural feature of this system is the presence of three spatially close electrophilic centers (the carbonyl group and two carbon atoms of the triple bond) in the substrate and several (two or three) nucleophilic atoms in the reagent. The combination of several nucleophilic and electrophilic centers accounts for the multichannel reactivity. The cyclizations are sensitive to the nature of nucleophile and can be directed towards the formation of different polycyclic heterocycles.

**Scheme 1:**
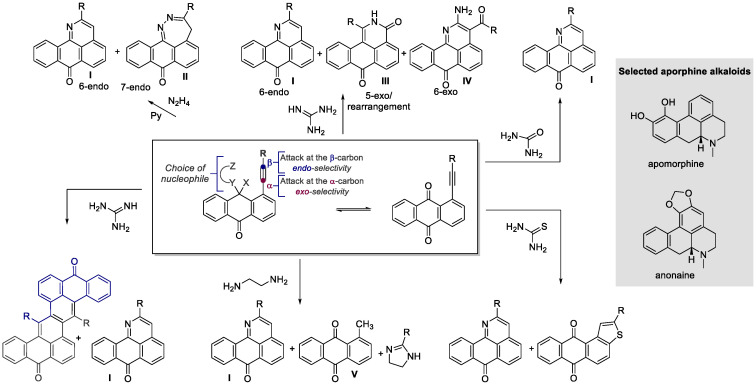
The variety of cyclization pathways in reactions of multifunctional nucleophiles with 1-(R-ethynyl)-9,10-anthraquinones. Selected aporphine alkaloids are shown in the grey box.

Interestingly, despite the different nature of the multifunctional nucleophiles, along with the formation of diverse new condensed systems, these reactions always provided at least some (and sometimes, exclusively) 2-R-7*H*-dibenzo[*de,h*]quinolin-7-ones I, i.e., the heterocyclic core of an important group of alkaloids of the *Aporphinoid* series (Scheme 1).

Taking into account the broad range of possible transformations and the close relationship of the resulting heterocycles to the *Aporphinoid* alkaloids [1,2,3,4,5], it was interesting to study the cyclization directions of 1-(R-ethynyl)-9,10-anthraquinones with other multi-centered nucleophiles related to urea and guanidine―acetamidine and phenylamidine.

## 2. Results and Discussion

### 2.1. Synthesis

**Scheme 2:**
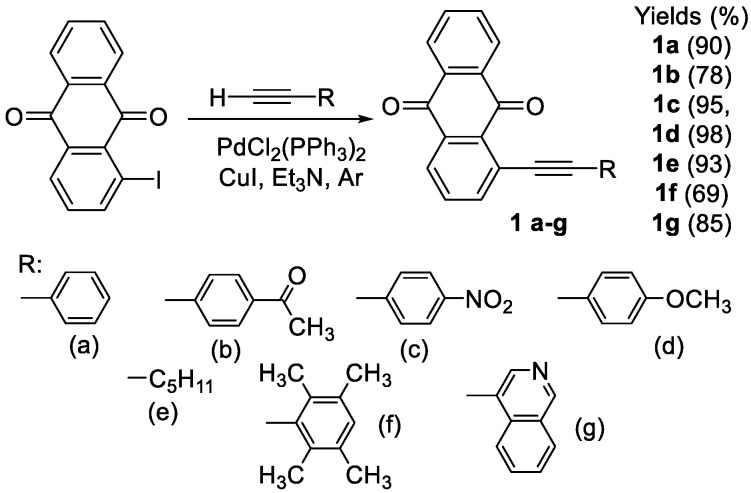
Synthesis of *peri*-R-ethynyl-9,10-anthraquinones. All reported yields in all schemes are isolated unless noted otherwise.

The starting 1-ethynylaryl-9,10-anthraquinones **1a**–**g** were prepared in 78–98% yields through Sonogashira cross-coupling of 1-iodo-9,10-anthraquinone with corresponding terminal alkynes, using the Pd(PPh_3_)_2_Cl_2_-CuI-Et_3_N catalytic system (Scheme 2).

**Scheme 3:**
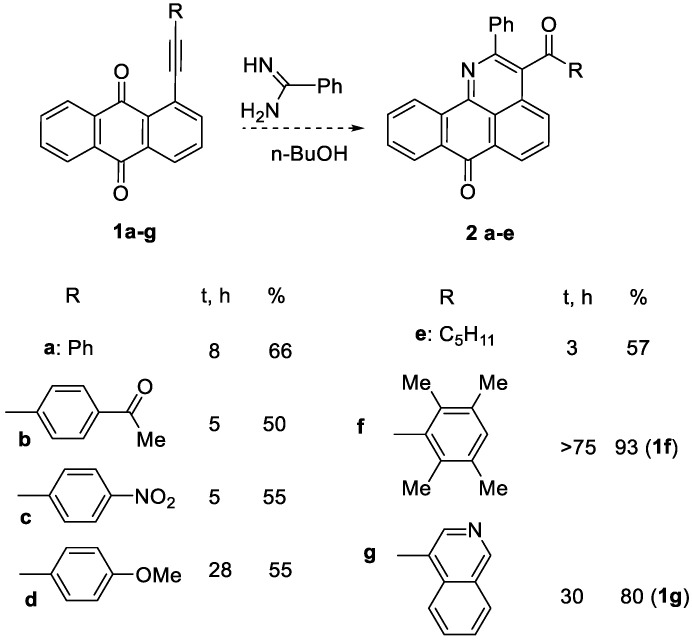
Interaction of phenylamidine with *peri*-R-ethynyl-9,10-anthraquinones.

It was found that the reaction of *peri*-R-ethynyl-9,10-anthraquinones with a 10-fold excess of benzamidine in boiling butanol (3–28 h) resulted in 2-R-3-aroyl-7*H*-dibenzo[*de,h*]quinolin-7-ones **2**, isolated in 50–66% yields (Scheme 3).

The formation of 2-R-3-aroyl-7*H*-dibenzo [*de,h*]quinolin-7-ones **2** agrees well with the concept of using alkynes as synthetic analogs of carbonyl compounds [6]. The relatively low yields of **2a**–**e** (50–66%) can be attributed to amidine hydrolysis due to the long reaction time. Not only did the benzamidine hydrochloride hydrate reactant contain water, but additional water was formed in the processes of neutralization and condensation. Indeed, benzamide was isolated as a side product in 32% in the case of **2a**. Note that alkynes **1f** and **1g** were unreactive towards benzamidine under these conditions. Only the initial acetylenyl anthraquinones **1f** (93%) and **1g** (80%) were isolated. Likely, the reaction was prohibited by the steric bulk introduced by the substituent at the alkyne.

Interestingly, the reaction of benzamidine with the alkyne substrate bearing a highly donor p-NH_2_Ph substituent had a much lower conversion lead to a mixture of products where the heterocyclic ketone **2** was detected in only <2% yield.

**Scheme 4:**
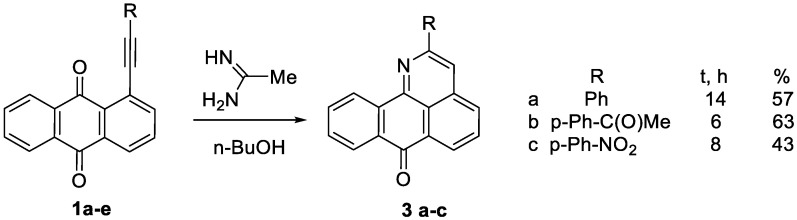
Interaction of acetamidine with *peri*-R-ethynyl-9,10-anthraquinones.

On the other hand, the reaction of the *peri*-R-ethynyl-9,10-anthraquinones **1** with acetamidine proceeded in a different direction. In this case, 2-R-7*H*-dibenzo[*de,h*]quinolin-7-ones (**3a**–**c**) were formed in 43–63% upon the reflux of the starting reagents in n-butanol (Scheme 4).

**Scheme 5:**
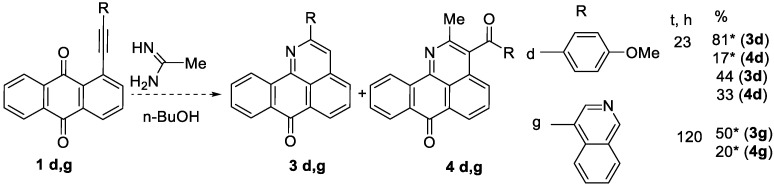
Non-selective reactions with acetamidine. * According to GS-MS analysis of the reaction mixture.

Interestingly, the reaction of acetamidine with **1d** and **1g** proceeded to form a mixture of both 2-methyl-3-(R-benzoyl)-7*H*-dibenzo[*de,h*]quinolin-7-ones (**4d**,**g**) and 2-R-7*H*-dibenzo[*de,h*]-quinolin-7-ones (**3d**,**g**) (Scheme 5).

**Scheme 6:**
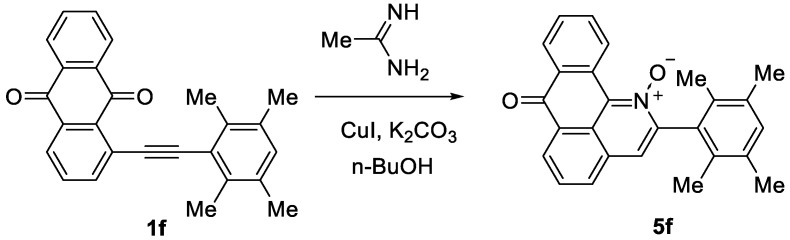
The unexpected formation of N-oxide in the cyclization of the durene-substituted alkyne.

The ratio of products **4d**:**3d** was 1:5, and **4g**:**3g**—2:5. Intriguing is the result of the reaction of the durene derivative **1f** with acetamidine (Scheme 6). Unexpectedly, this reaction led to the formation of an N-oxide **5f** (62.5%). Note that the same durene derivative did not interact with benzamidine. This is consistent with the importance of steric factors in the cyclization (acetaminidine is less spatially hindered than benzamidine).

The N-oxide ^1^H and ^13^C NMR peaks were shifted to the strong fields relative to the peaks of their analogs without the N-O bond. This suggests that the oxygen atom of the N-oxide moiety serves as an electron donor towards the acceptor N-heterocyclic moiety. The IR-spectrum contains characteristic N-O peaks at 1249 and 1313 cm^−1^. The N-oxide is a relatively polar dark-red compound.

**Scheme 7:**
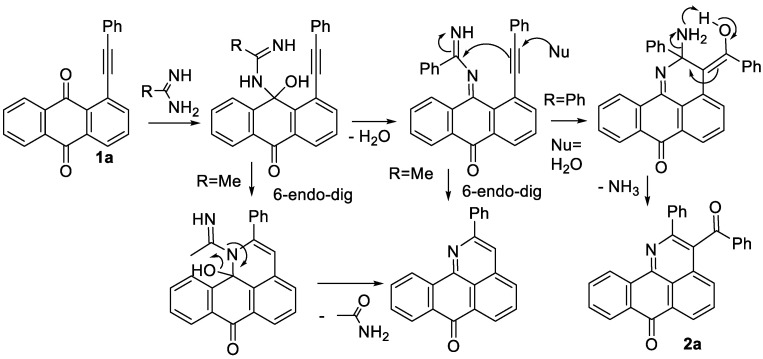
The general scheme for the formation of the two types of tetracyclic heterocyclic cores from R-ethynyl-9,10-anthraquinones.

Structures of the isolated products suggest that di(benzo)quinoline skeleton formation can proceed by two pathways illustrated in Scheme 7: either an intramolecular addition of the amidine nitrogen atom, followed by fragmentation (for R=Me) or cyclization–fragmentation with the participation of an external nucleophile, e.g., water (for R=Ph). These scenarios are consistent with the isolation of the fragmentation amide product.

The observed transformations, i.e., the formation of aroyl derivatives, are well consistent with the concept of alkynes as chemical equivalents of carbonyls, described in our recent review [6].

The resulting compounds are of considerable interest for medical chemistry, due to the relation of the 7*H*-dibenzo[*de,h*]quinoline-7-one skeleton to natural alkaloids of the *Isoaporphinoid* series. Considering this relation, we explored biological activity of the new heterocyclic compounds.

### 2.2. Pharmacological Study

**Scheme 8:**
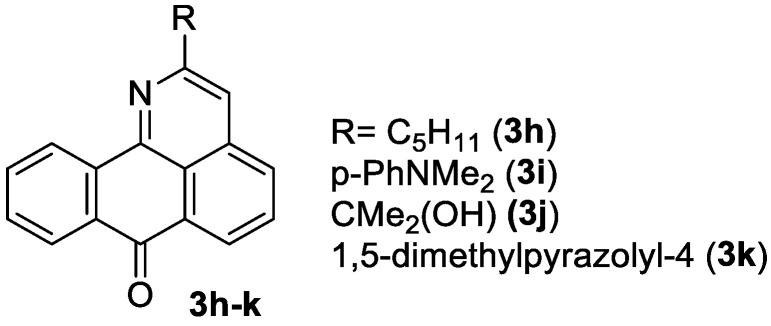
Synthesis of the tested compounds (**3h**–**k**).

For the pharmacological study, we additionally synthesized a series of 2-R-7*H*-dibenzo[*de,h*]quinolin-7-ones (**3h**–**k**) using the method previously described by us [3,4] (Scheme 8).

The pharmacological effects of 2-R-7H-dibenzo[*de,h*]quinolin-7-ones (**2**–**3**) were studied. The preliminary computer SAR analysis showed that the tested compounds had high probability of immunomodulative (0.537–0.746), anti-ankylosing spondylitis (0.537–0.702), and antineoplastic (0.510–0.839) activities. Based on these predictions, studies of their anti-inflammatory and cytotoxic activity were carried out. Additionally, the compounds were verified at the binding site of the intended targets.

### 2.3. Anti-Inflammatory Activity

The anti-inflammatory activity of the synthesized compounds was investigated in vivo in comparison with indomethacin on two models of local inflammation induced by histamine or concanavalin A injection. The last one was used as the inductor of allergic-like inflammation, owing to its immunogenic activity. The results were expressed as values of inflammatory edema indexes (IEI) and percentages of anti-inflammatory activity (AIA) relative to the control group (Table 1).

In the histamine-induced model, the tested quinolinones showed pronounced anti-inflammatory activity at a dose of 20 mg/kg i.p. comparable to that of the nonsteroidal anti-inflammatory drug indomethacin (70–125% relative to indomethacin). In the series of derivatives, the most potent effect was observed for compound **3a**, while **3j** alone had an insignificant effect (16% reduction in edema versus control) (Table 1).

In the model of concanavalin A-inflammation, double intraperitoneal administration of the same agents to mice at a dose of 20 mg/kg increased the anti-inflammatory effect with the exception of **7**, where the effect was insignificant (18% against control). The agent **3d** showed the greatest activity, which was significantly higher than that of indomethacin (by 1.5 times). Derivatives **3a**, **3d**, **3h**, and **3i** showed the same tendency, increasing the activity above that of reference drug by 1.3–1.4 times (Table 1).

### 2.4. Cytotoxicity

The cytotoxicity study of the quinolinones **2**–**3** was evaluated in vitro by MTT assay on immortalized humane fibroblasts (hTERT), humane breast cancer cell line (MCF7) and humane glioblastoma cell line (U-87MG and) versus doxorubicin as a reference compound.

All tested molecules exhibited much more low cytotoxicity than doxorubicin, and several quinolinones showed cytotoxic activity in cervical cancer culture (HeLa). Compounds **3j** and **3k** had a moderate effect (IC_50_ = 21.3 μM) that was 3.5 times lower than doxorubicin, and **2** had a weak cytotoxicity (IC_50_ = 86.3 μM, which was 14 times worse than the reference). In addition, compound **3j** also revealed cytotoxic properties in multiform glioblastoma (U-87 MG, IC_50_ = 50.1 μM,) and breast cancer (MCF7, IC_50_ = 92.9 μM), which were significantly less than those of doxorubicin (Table 2). It should be noted that the abovementioned quinolinones **3a**, **3j**, and **3k** did not have a cytotoxic effect on immortal fibroblasts, which indicates their selective antitumor activity.

### 2.5. Molecular Docking

To test the assumption of possible intercalation of new polyaromatic compounds into the DNA molecule as a mechanism of their antitumor action, by analogy with cytotoxic antibiotics such as doxorubicin, molecular docking of the new compounds to the doxorubicin binding site in DNA was performed. Intercalation is possible if the ligand is of suitable size and chemical properties and can fit between the bases of the DNA. Such ligands usually have a polycyclic, aromatic structure, and are flat, which is observed for new compounds. The results of the docking scoring function are ranked in Table 3.

**Figure 1:**
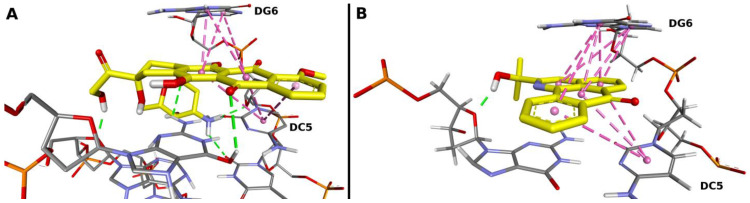
(**A**)—intercalation of doxorubicin into DNA, (**B**)—intercalation modeling for **3j**. Ligands are yellow, non-covalent interactions are shown by dashed lines: pink—stacking interactions, green—hydrogen bonds.

Doxorubicin is characterized by low binding energy (−12.4 kcal/mol) with DNA molecules, determined by a large number of stacking interactions of the pi-systems of anthraquinone aglycone and purine and pyrimidine bases of DNA, as well as the active formation of hydrogen bonds between the polar atoms of doxorubicin and DNA. Among the new molecules, **3j** should be noted, whose binding energy (−7.1 kcal/mol) was significantly lower than the other molecules (Figure 1).

The modeling of the intercalation of new compounds in DNA shows the theoretical possibility of its entering between cytosine and guanine with the active formation of stacking interactions of pi-systems. This type of binding may be a possible molecular mechanism of the antitumor effect of new derivatives.

## 3. Experimental

Melting points were determined with a Kofler apparatus. The IR-spectra were recorded in KBr pellets on a ‘Bruker Vector 22’ spectrometer. NMR spectra were recorded on a ‘Bruker AV-400’ 400.13 MHz (^1^H) or DX-500 500.13 MHz (^1^H) and 100.61 MHz (^13^C) in CDCl3. ^13^C spectra were recorded with complete suppression of the ^13^C-^1^H spin–spin interaction on a pulsed J-modulation program. Mass spectra were obtained on a Finnigan SSQ-710 mass-spectrometer by the direct injection method (the temperature of the ionization chamber was 220–270 °C, the ionization voltage, 70 eV). Column chromatography was performed on Al_2_O_3_. Chromato-mass analysis was performed using an Agilent GCMS 6890N/5973N spectrometer with quartz capillary column DB (10.25 mm, 30 m, coating 2.5 μm polysiloxane), evaporator temperature 250 °C, column temperature―initial 50 °C 2 min, heating 10 °C/min to 250 °C, then 250 °C 100 min. Total time of analysis was 122 min. The carrier gas was helium, flow 1 mL/min. TLC monitoring was carried out on Silufol 60 F254 (Merck, 0.2 mm) plates. Benzo- and acetamidines, PdCl_2_(PPh_3_)_2_, CuI, and Et_3_N were commercially available reactants.

### 3.1. Preparation of 1-(R-Ethynyl)-9,10-antraquinones (***1a**–**g***)

A mixture of 1-iodo-9,10-anthraquinone (4.5 mmol) and corresponding 1-alkyne (4.5 mmol), CuI (0.027 mmol), PdCl_2_(PPh_3_)_2_ (0.013 mmol), and Et_3_N (12.7 mmol) in 50 mL of toluene was stirred under an argon atmosphere at 65 °C (2–4 h) until iodide was consumed (TLC control). The reaction mixture was cooled and filtered through SiO_2_ (25 × 30 mm) using toluene as the eluent. The solvents were evaporated, and subsequent recrystallization gave pure compounds **1a**–**g**.

*1-Phenylethynyl-9,10-anhraquinone* (**1a**). The yield was 1.25 g (90%), m.p. 159–160 °C (from toluene), m.p. (lit) 159–160 °C [7].

*1-[2-(4-Acetylphenyl)ethynyl]-9,10-anthraquinone* (**1b**). The yield was 1.22 g (78%), m.p. 216–217 °C (from toluene-ethyl acetate), m.p. (lit) 217–218 °C [8].

*1-[2-(4-Nitrophenyl)ethynyl]-9,10-anthraquinone* (**1c**). The yield was 1.52 g (95%), m.p. 214–215 °C (from toluene), m.p. (lit) 214–215 °C [7].

*1-[2-(4-Methoxyphenyl)ethynyl]-9,10-anthraquinone* (**1d**). The yield was 1.49 g (98%), m.p. 194–195 °C (from toluene), m.p. (lit) 193–194 °C [7].

*1-(Heptyn-1-yl)-9,10-anthraquinone* (**1e**). The yield was 1.26 g (93%), m.p. 62–64 °C (from hexane), m.p. (lit) 62–63 °C [3].

*1-[2-(2,3,5,6-Tetramethylphenyl)ethynyl]-9,10-anthraquinone* (**1f**). The yield was 1.02 g (69%), time of reaction 5 h, m.p. 246–247 °C (from toluene-ethyl acetate). HRMS, Found: *m*/*z*: 364.1456 [M]^+^. C_26_H_20_O_2_. Calcd.: M = 364.1458. IR, cm^−1^, ν: 1668 (C=O), 2191 (C≡C). ^1^H NMR (CDCl_3_, 500 MHz) δ 2.28 (s, 6H, C_m_-CH_3_), 2.63 (s, 6H, C_o_-CH_3_), 6.97 (s, 1H, H_p_), 7.72–7.83 (m, 3H, H-3, H-7, H-6), 8.01(d, 1H, *J* = 10 Hz, H-2), 8.29–8.33 (m, 2H, H-4, H-5), 8.37 (d, 1H, *J* = 10 Hz, H-10). ^13^C NMR (CDCl_3_, 100 MHz) δ 17.7, 19.9, 95.8, 96.3, 123.1, 124.5, 126.8, 126.9, 127.5, 127.8, 132.1, 132.6, 132.7, 132.8, 133.5, 133.6, 134.3, 134.5, 137.1, 140.3, 181.7, 183.0.

*1-[2-(Iso-quinolin-3-yl)ethynyl]-9,10-anthraquinone* (**1g**). The yield was 1.37 g (85%), m.p. 224–226 °C (from toluene), m.p. (lit) 223–224 °C [8].

### 3.2. Reaction of 1-(R-Ethynyl)-9,10-anthraquinones (***1a**–**g***) with Benzamidine Hydrochloride Hydrate

A mixture of 1-(R-ethynyl)-9,10-anthraquinone (0.5 mmol), benzamidine (5 mmol), CuI (0.02 mmol), and K_2_CO_3_ (5 mmol) boiled in 10 mL of 1-butanol (3–28 h) (TLC control). The reaction mixture was cooled. The solvent was removed in vacuo, and residue was purified by column chromatography (toluene, ethyl acetate). Subsequent recrystallization gave pure compounds **2a**–**e**.

**Scheme 9:**
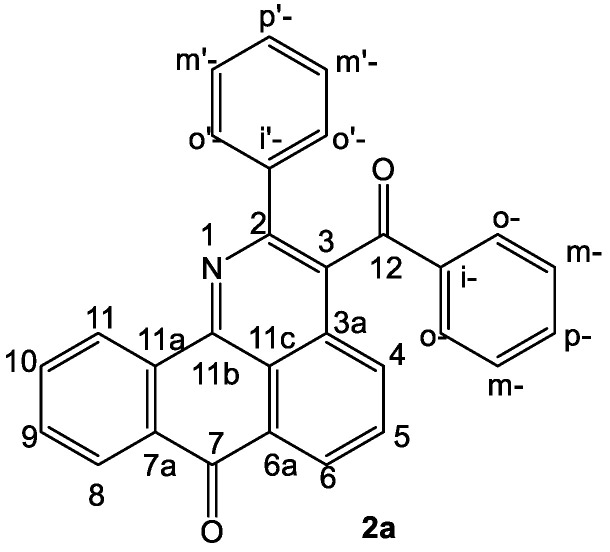
Atom numbering for compound **2a**.

The yield of *2-benzo-3-benzoyl-7H-dibenzo[de,h]quinolin-7-one* **2a** (Scheme 9) was 66%, time of reaction 8 h, m.p. 276–277 °C (from toluene). HRMS, Found: *m*/*z* 411.1248 [M]^+^. C_29_H_17_NO_2_. Calcd.: M = 411.1259. IR, cm^−1^, ν: 1593, 1658 (C=O). ^1^H NMR (CDCl_3_, 500 MHz) δ 7.28–7.34 (m, 5H, H_o−_, H_m_, H_p_), 7.44 (t, 1H, *J* = 5 Hz, H-5) 7.69–7.78 (m, 5H, H_o’_, H_p’_, H_m’_), 7.84–7.91 (m, 2H, H-10, H-9), 8.13 (d, 1H, *J* = 10 Hz, H-4), 8.48 (d, 1H, *J* = 10 Hz, H-6), 8.70 (d, 1H, *J* = 10 Hz, H-8), 9.09 (d, 1H, *J* = 10 Hz, H-11). ^13^C NMR (CDCl_3_, 100 MHz) δ 121.5, 126.1, 127.8, 128.5, 128.7, 129.0, 129.0, 129.4, 129.8, 130.0, 131.1, 131.5, 131.6, 132.8, 134.0, 134.2, 134.3, 136.6, 137.6, 139.7, 149.5, 151.0, 183.5, 197.7. Anal. Calcd for C_29_H_17_NO_2_: C, 84.65; H, 4.16; N, 3.40. Found: C, 84.58; H, 4.05; N, 3.39.

The yield of benzamide was 32%, m.p. 127.5–128.5 °C (from toluene-ethyl acetate), m.p. (lit) 128–130 °C [9].

The yield of *2-benzo-3-(p-acetylbenzoyl)-7H-dibenzo[de,h]quinolin-7-one* **2b** was 49.5%, time of reaction 5 h, m.p. 312–313 °C (from toluene). HRMS, Found: *m*/*z* 453.1357 [M]^+^. C_31_H_19_NO_3_. Calcd.: M = 453.1360. IR, cm^−1^, ν: 1591, 1660, 1685 (C=O). ^1^H NMR (CDCl_3_, 500 MHz) δ 2.55 (s, 3H, -CH_3_), 7.29–7.34 (m, 3H, H-5, H_m_), 7.72–7.75 (m, 5H, H_m’,_ H_o’,_ H_p’_), 7.81 (d, 2H, H_o,_ *J* = 10), 7.87 (t, 1H, *J* = 10, H-10), 7.93 (t, 1H, *J* = 10 Hz, H-9), 8.14 (d, 1H, *J* = 10 Hz, H-4), 8.47 (d, 1H, *J* = 10 Hz, H-6), 8.72 (d, 1H, *J* = 10 Hz, H-8), 9.09 (d, 1H, *J* = 10 Hz, H-11). ^13^C NMR (CDCl_3_, 100 MHz) δ 26.7, 121.3, 125.9, 127.6, 128.0, 128.2, 128.4, 129.0, 129.3, 129.6, 129.8, 129.8, 130.9, 131.0, 131.6, 132.5, 133.8, 134.1, 136.2, 139.3, 140.3, 140.4, 149.7, 151.1, 183.1, 196.9, 197.1. Anal. Calcd for C_31_H_19_NO_3_: C, 82.10; H, 4.22; N, 3.09. Found: C, 82.15; H, 4.09; N, 3.16.

The yield of *2-benzo-3-(p-nitrobenzoyl)-7H-dibenzo[de,h]quinolin-7-one* **2c** was 55%, time of reaction 5 h, m.p. 324–325.5 °C (from toluene). HRMS, Found: *m*/*z* 456.1115 [M]^+^. C_29_H_16_N_2_O_4_. Calcd.: M = 456.1105. IR, cm^−1^, ν: 1348, 1525 (NO_2_), 1591, 1658 (C=O). Low solubility in CHCl_3_ and DMSO. ^1^H NMR (CDCl_3_, 400 MHz) δ 7.28–7.35 (m, 3H, H-5, H_m’_), 7.70–7.80 (m, 5H, H_p’_, H_o’_, H_o_) 7.87 (t, 1H, *J* = 8, H-9), 7.97 (t, 1H, *J* = 8, H-10), 8.08 (d, 2H, *J* = 8 Hz, H_m_), 8.19 (d, 1H, *J* = 8 Hz, H-4), 8.49 (d, 1H, *J* = 8 Hz, H-6), 8.74 (d, 1H, *J* = 8 Hz, H-8), 9.09 (d, 1H, *J* = 8 Hz, H-11).

The yield of *2-benzo-3-(p-methoxybenzoyl)-7H-dibenzo[de,h]quinolin-7-one* **2d** was 55%, time of reaction 28 h, m.p. 279–280 °C (from toluene). HRMS, Found: *m*/*z* 441.1362 [M]^+^. C_30_H_19_NO_3_. Calcd.: M = 441.1360. IR, cm^−1^, ν: 1595, 1658 (C=O). ^1^H NMR (CDCl_3_, 400 MHz) δ 3.79 (s, 3H, -CH_3_), 6.76 (d, 2H, *J* = 8 Hz, H_m_), 7.32–7.37 (m, 3H, H-5, H_o_), 7.68–7.73 (m, 3H, H_o’_, H_p’_), 7.80–7.90 (m, 4H, H-10, H-9, H_m’_), 8.11 (d, 1H, *J* = 8 Hz, H-4), 8.47 (d, 1H, *J* = 8 Hz, H-6), 8.68 (d, 1H, *J* = 8 Hz, H-8), 9.09 (d, 1H, *J* = 8 Hz, H-11). ^13^C NMR (CDCl_3_, 100 MHz) δ 55.6, 114.1, 1215, 126.0, 127.7, 128.5, 129.0, 129.3, 129.3, 129.8, 129.9, 130.8, 131.0, 131.5, 131.7, 132.3, 132.7, 134.2, 134.3, 136.6, 139.7, 149.2, 150.5, 164.3, 183.5, 196.1. Anal. Calcd for C_30_H_19_NO_3_: C, 81.62; H, 4.34; N, 3.17. Found: C, 81.11; H, 4.22; N, 3.38.

The yield of *2-benzo-3-hexanoyl-7H-dibenzo[de,h]quinolin-7-one* **2e** was 57%, time of reaction 3 h, m.p. 178.5–179 °C (from hexane). HRMS, Found: *m*/*z* 405.1732 [M]^+^. C_28_H_23_NO_2_. Calcd.: M = 405.1723. IR, cm^−1^, ν: 1656, 1689 (C=O). ^1^H NMR (CDCl_3_, 400 MHz) δ 0.78 (t, 3H, -CH_3_), 1.04–1.16 (m, 4H, -CH_2_-CH_2_-Me), 1.53 (t, 2H, -CH_2_-Pr), 2.37 (t, 2H, -CH_2_-Bu), 7.52–7.57 (m, 3H, H-5, H_m’_), 7.69 (t, 1H, *J* = 8 Hz, H-10), 7.81–7.85 (m, 3H, H_o’_, H_p’_), 7.96 (t, 1H, *J* = 8 Hz, H-9), 8.21 (d, 1H, *J* = 8 Hz, H-4), 8.44 (d, 1H, *J* = 8 Hz, H-6), 8.69 (d, 1H, *J* = 8 Hz, H-8), 9.04 (d, 1H, *J* = 8 Hz, H, H-11). ^13^C NMR (CDCl_3_, 100 MHz) δ 13.9, 22.3, 23.9, 31.17, 45.2, 121.5, 126.1, 127.7, 129.0, 129.4, 129.5, 129.91, 131.0, 131.0, 132.6, 131.7, 132.8, 134.2, 136.4, 139.8, 149.1, 150.2, 183.5, 208.2.

### 3.3. Reaction of 1-(R-Ethynyl)-9,10-anthraquinones (***1a**–**g***) with Acetamidine Hydrochloride

**Scheme 10:**
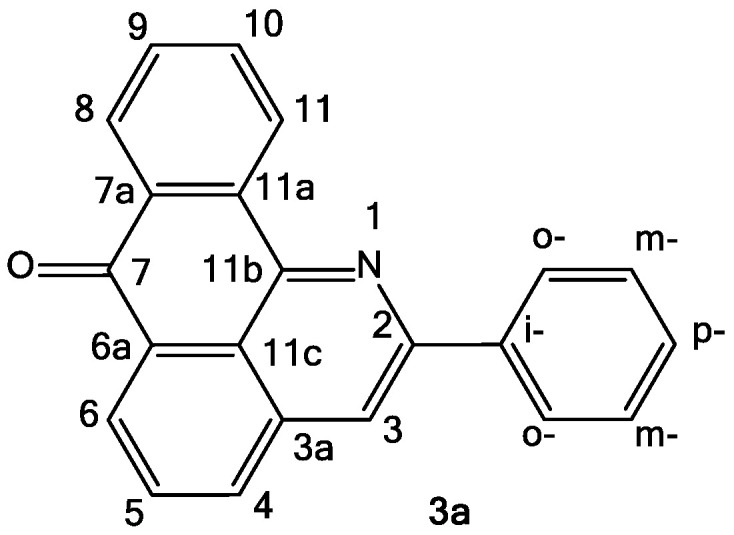
Atom numbering for compound **3a**.

A mixture of 1-(R-ethynyl)-9,10-anthraquinone (0.5 mmol), acetamidine (5 mmol), CuI (0.02 mmol), and K_2_CO_3_ (5 mmol) boiled in 10 mL of 1-butanol (TLC control). The reaction mixture was cooled. The solvent was removed in vacuo, and residue was purified by column chromatography (toluene, ethyl acetate). Subsequent recrystallization gave pure compounds **3a**–**c** (Scheme 10) and **5f**.

The yield of *2-phenyl-7H-dibenzo[de,h]quinolin-7-one* **3a** was 57%, time of reaction 14 h, m.p. 207–208 °C (recrystallization from toluene), m.p. (lit) 207–208 °C [1].

The yield of *2-(p-acetylphenyl)-7H-dibenzo[de,h]quinolin-7one* **3b** was 63%, time of reaction 6 h, m.p. 324–325 °C (from toluene). HRMS, Found: *m*/*z* 349.1094 [M]^+^. C_24_H_15_NO_2_. Calcd.: M = 349.1103. IR, cm^−1^, ν: 1595, 1658 (C=O). ^1^H NMR (CDCl_3_, 400 MHz) δ 2.72 (s, 3H, -CH_3_), 7.72 (t, 1H, *J* = 8 Hz, H-9), 7.89 (t, 1H, *J* = 8 Hz, H-10), 7.97 (t, 1H, *J* = 8 Hz, H-5), 8.18 (m, 2H, *J* = 8 Hz, H_o_), 8.25–8.29 (m, 2H, H-4, H-3), 8.43–8.48 (m, 3H, H-8, H_m_), 8.69 (d, 1H, *J* = 8 Hz, H-6), 9.13 (d, 1H, *J* = 8 Hz, H-11). ^13^C NMR (CDCl_3_, 100 MHz) δ 26.65, 117.3, 122.2, 125.4, 127.0, 127.5, 128.8, 129.0, 129.9, 130.5, 130.8, 132.4, 133.7, 133.9, 1359, 136.5, 137.0, 143.2, 148.5, 150.2, 183.2, 193.1.

The yield of *2-(p-nitrophenyl)-7H-dibenzo[de,h]quinolin-7-one* **3c** was 43%, time of reaction 8 h, m.p. 295–296 °C (from 1,4-dioxane), m.p. (lit) 294–295 °C [3].

The yield of *2-(p-methoxyphenyl)-7H-dibenzo[de,h]quinolin-7-one* **3d** was 44%, time of reaction 23 h, m.p. 203–204 °C (from toluene), m.p. (lit) 206–207 °C [3].

The yield of *2-methyl-3-(p-methoxybenzoyl)-7H-dibenzo[de,h]quinolin-7-one* **4d** was 33%, time of reaction 23 h, m.p. 232.5–233.5 °C (from toluene). HRMS, Found: *m*/*z* 379.1203 [M]^+^. C_25_H_17_NO_3_. Calcd.: M = 379.02. IR, cm^−1^, ν: 1595, 1649 (C=O). ^1^H NMR (CDCl_3_, 500 MHz) δ 2.68 (s, 3H, CH_3_), 3.89 (s, 3H, -OCH_3_), 6.95 (d, 2H, *J* = 10 Hz, H_m_), 7.70 (t, 1H, *J* = 10 Hz, H-5), 7.79–7.89 (m, 5H, H-4, H-9, H-10, H_o_), 8.45 (d, 1H, *J* = 10 Hz, H-6), 8.62 (d, 1H, *J* = 10 Hz, H-8), 9.01 (d, 1H, *J* = 10 Hz, H-11). ^13^C NMR (CDCl_3_, 100 MHz) δ 23.3, 55.8, 67.9, 114.6, 125.8, 127.8, 129.4, 130.7, 130.9, 131.1, 131.3, 132.6, 132.7, 133.7, 134.3, 136.7, 149.4, 164.9, 183.1, 195.7.

The yield of *1-N-oxide-2-(2,3,5,6-tetramethylphenyl)-7H-dibenzo[de,h]quinolin-7-one* **5f** was 62.5%, time of reaction 70 h, m.p. 232.5 °C (from methanol). HRMS, Found: *m*/*z* 379.1562 [M]^+^. C_26_H_21_NO_2_. Calcd.: M = 379.1567. IR, cm^−1^, ν: 1280 (N→O), 1571 (C=O). ^1^H NMR (CDCl_3_, 500 MHz) δ 2.17 (s, 6H, C_m_-CH_3_), 2.35 (s, 6H, C_o_-CH_3_), 6.75 (d, 1H, *J* = 10 Hz, H-4), 7.22 (s, 1H, H-3), 7.40–7.44 (m, 2H, H-5, H-10), 7.49 (t, 1H, *J* = 10 Hz, H-9), 8.14 (d, 1H, *J* = 10 Hz, H-6), 8.45–8.49 (m, 2H, H-8, H-11). ^13^C NMR (CDCl_3_, 100 MHz) δ 16.4, 19.7, 123.5, 124.7, 125.2, 126.2, 126.5, 126.6, 127.4, 127.5, 128.6, 129.1, 129.3, 130.2, 132. 3, 132.7, 133.1, 134.5, 140.3, 183.8, 188.3.

### 3.4. Animal Studies

The pharmacological studies were carried out on outbred albino male mice and C57BL/6 female mice weighing 20–25 g with eight animals in each group. All animals were taken from SPF-vivarium of the ICG SB RAS (Institute of Cytology and Genetics of Russian Academy of Sciences Siberian Branch). Mice were housed in wire cages at 22–25 °C on a 12 h light–dark cycle. The animals had free access to a standard pellet diet, and tap water was available ad libitum. All experimental procedures were approved by the Bio-Ethical Committee of Medicine Chemistry Department of Novosibirsk Institute of Organic Chemistry SB RAS (Protocol Code P-1-03.2020-14) in accordance with the European Convention for the Protection of Vertebrate Animals used for Experimental and other Scientific Purposes, and the requirements and recommendations of the Guide for the Care and Use of Laboratory Animals.

### 3.5. Anti-Inflammatory Activity

The pharmacological studies were carried out on outbred albino male mice and C57BL/6 female mice weighing 20–25 g with eight animals in each group. The anti-inflammatory activity of compounds **2**–**3** was evaluated in the histamine-induced and concanavalin A-induced mouse paw edema models. The test compounds were dissolved in saline containing 0.5% Tween 80 just before intraperitoneal administration at the dose of 20 mg/kg. The reference agent indomethacin (‘Fluka BioChemica’) was administered by the same method and dose. The control group of animals received an equivalent volume of water–Tween mixture.

### 3.6. Histamine-Induced Mouse Paw Edema Model

The 8 outbred male albino mice were subplantar injected into the hind paw with 0.05 mL 0.01% histamine (“Sigma Aldrich”, Saint Louis, MO, USA) in water solution. The tested compounds were administered 1 h before the histamine injection. The animals were sacrificed by cervical dislocation 5 h after the phlogogen injection.

### 3.7. Concanavaline A-Induced Mouse Paw Edema Model

The 8 female mice C57Bl/6 were subplantar injected into the hind paw with 0.02 mL 0.5% concanavaline A (“Sigma Aldrich”) aqueous solution. The test compounds were administered twice 24 h and 1 h before phlogogen. The animals were sacrificed by cervical dislocation 1 h after the phlogogen injection.

Both hind paws were cut off at the ankle joint and weighed. The ratio of the difference in weight between the treated and untreated hind paws to the weight of the untreated hind paw was used as an inflammatory edema index (IEI). The relative percentage of IEI versus control IEI (100%) was calculated for each test group. The anti-inflammatory activity (AIA) was presented as a difference between 100% in the control group and relative percentage of IEI in the test group.

### 3.8. Cytotoxicity Assay

Cell lines were obtained from the American Type Culture Collection: U-87 MG (ATCC number HBT-14), MCF7 (ATCC number HTB-22), A549 (ATCC number CRM-CCL), HeLa (ATCC number CCL-2). Immortalized human fibroblasts hTERT (as noncancerous control) were obtained from ICG SB RAS (Novosibirsk, Russia). DMEM with 10% fetal bovine serum (Gibco, Amarillo, TX, USA) was used for culturing. Optical density was measured on a Multiskan RC spectrophotometer (LabSystems, Vantaa, Finland). The MTT test was carried out according to the standard procedure [10] using the reagent 3-(4,5-dimethylthiazol-2-yl)-2,5-diphen-yltetrazolium bromide (MTT, Sigma). Cells were seeded in 96 well plates at 5000 cells/well and incubated for 24 h at 37 C in 5% CO_2_. After 24 h, various concentrations of test compounds (10, 25, 50, 100 µm) in DMSO were added and the cells were incubated for an additional 72 h. The viability for DMSO (negative control) was calculated as 100%. All culture experiments were carried out independently three times with two repeats in the experiment, and the final result is presented as concentration, which causes 50% inhibition of cell population growth (IC_50_ ± SEM).

### 3.9. Molecular Docking

Molecular modeling was carried out in the Schrodinger Maestro visualization environment using applications from the Schrodinger Small Molecule Drug Discovery Suite 2016-1 package [Schrödinger, LLC, New York, NY, USA, 2016]. Three-dimensional structures of the molecules were obtained in the LigPrep application using the OPLS3 force field [11]. All possible tautomeric forms of compounds, as well as various states of polar protons of molecules in the pH range of 7.0 ± 2.0, were taken into account. For the calculations, the XRD model of DNA with intercalated doxorubicin with PDB ID 1D12 (resolution 1.7 Å) was chosen [12]. To investigate DNA binding, molecular docking of new compounds was performed at the binding site of doxorubicin in the Glide application. The search area for docking was selected automatically, based on the size and physico-chemical properties of doxorubicin. The extra precision (XP) algorithm of docking was applied. Docking was performed in comparison with doxorubicin. The three-dimensional structure of doxorubicin was obtained in the PubChem database and prepared in the *LigPrep* application. Non-covalent interactions of compounds in the binding site were visualized using Biovia Discovery Studio Visualizer 2016 [Dassault Systèmes BIOVIA, San Diego, CA, USA, 2016].

### 3.10. Statistical Analysis

Data represent mean ± standard error (SEM) from groups of animals. Statistical analysis was applied with parametric and non-parametric methods using “STATISTICA 6” software. The differences were significant at *p* < 0.05.

## 4. Conclusions

The list of multifunctional nucleophiles capable of transforming *peri*-R-ethynyl-9,10-anthraquinones into tetracyclic products was extended to include acetamidine and phenylamidine. These nucleophiles induce cascade transformations that include a sequence of addition/elimination/cyclization steps that yield 2-R-7*H*-dibenzo[*de,h*]quinolin-7-ones and(or) 2-R-3-aroyl-7*H*-dibenzo[*de,h*]quinolin-7-ones systems. These processes expand the range of known peri-cyclizations, the relatively sparse class of reactions that receive considerable interest for building polycyclic systems, including fusion of additional cycles at the zigzag edge of carbon nanostructures [13,14,15,16,17,18].

The anti-inflammatory and antitumor properties of the new 2-R-7*H*-dibenzo[*de,h*]quinolin-7-ones (**2**–**3**) were investigated in vivo, in vitro, and in silico. The synthesized compounds exhibited high anti-inflammatory activity at dose 20 mg/kg i.p. in the models of exudative (histamine-induced) and immunogenic (concanavalin A-induced) inflammation. The derivatives **2b**, **2d**, **3b**, and **3i** had the most potent effects, which are comparable with those of indomethacin. All compounds had no cytotoxic effect on immortalized humane fibroblasts. Compounds **3j** and **3k** show moderate (IC50 = 21.3 μM) antitumor activity on cervical cancer culture HeLa. Compound **3j** also exhibited a weak antitumor effect on multiform glioblastoma and breast cancer cell cultures. Molecular docking data demonstrate that such molecules as **3j** can potentially intercalate into the binding site in the DNA molecule similarly to the reference antitumor drug doxorubicin.

## Figures and Tables

**Table 1:** Anti-inflammatory activity of tested substances in mouse paw edema models.

Agent	Dose, mg/kg	Histamine-Induced Inflammation		Concanavalin A-Induced Inflammation	
		IEI, %	AIA, %	IEI, %	AIA, %
Control	-	34.3 ± 2.6 ^###^	0	37.7 ± 2.8 ^##^	0
**9 2d**	20	23.0 ± 1.9 **	33	21.4 ± 1.6 ***	43
**8 2d**	20	22.7 ± 2.3 **	34	27.9 ± 1.2 **	26
**2 3a**	20	13.9 ± 0.8 ***	60	17.8 ± 1.8 ***	53
**1 3b**	20	22.1 ± 1.8 **	36	18.0 ± 2.0 ***	52
**4 3d**	20	19.9 ± 2.6 **	42	15.2 ± 1.6 ***^##^	60
**3 3h**	20	23.9 ± 1.7 **	30	19.7 ± 2.3 ***	48
**5 3i**	20	22.6 ± 2.3 **	34	18.5 ± 1.6 ***	51
**6 3j**	20	28.9 ± 2.0 ^##^	16	22.1 ± 2.5 **	41
**7 3k**	20	22.7 ± 1.7 **	34	31.1 ± 2.4 ^#^	18
Indomethacin	20	17.8 ± 2.7 ***	48	23.4 ± 2.2 **	38

** *p* < 0.01, *** *p* < 0.001 differences with control group are significant; ^#^ *p* < 0.05, ^##^ *p* < 0.01, ^###^ *p* < 0.0001 differences with reference group are significant.

**Table 2:** Cytotoxicity of quinolinones versus doxorubicin.

Agent	IC_50_ ± SEM, μM				
	hTERT	U-87 MG	A549	MCF7	HeLa
Doxorubicin	4.1 ± 1.0	2.3 ± 0.6	4.05 ± 0.9	3.6 ± 0.4	6.0 ± 1.0
**2b**	˃100	˃100	˃100	˃100	˃100
**2d**	˃100	˃100	˃100	˃100	˃100
**3a**	˃100	˃100	˃100	˃100	86.3 ± 12.9
**3b**	˃100	˃100	˃100	˃100	˃100
**3d**	˃100	˃100	˃100	˃100	˃100
**3h**	˃100	˃100	˃100	˃100	˃100
**3i**	˃100	˃100	˃100	˃100	˃100
**3j**	˃100	50.1 ± 5.9	˃100	92.9 ± 7.2	21.3 ± 5.9
**3k**	˃100	˃100	˃100	˃100	22.7 ± 7.3

**Table 3:** DNA docking results for new molecules.

Ligand	Binding Energy *, kcal/mol
Doxorubicin	−12.4
**3j**	−7.1
**3k**	−6.1
**3h**	−6.0
**2b**	−5.2
**3a**	−5.1
**3b**	−5.0
**3i**	−4.9
**3d**	−4.7
**2d**	−4.1

* Value is not genuine binding energy but an estimated docking score.

## Data Availability

Data regarding synthesis, isolation and characterization are available upon request from S.V. Information related to the biological activity studies is available from I.S.

## Sample Availability

Samples of the compounds may be available from the authors but only with the permission of the Institute of Chemical Kinetics and Combustion.
